# Supplementary material for: Prevalence of Essential Nutrient Supplement Use and Assessment of the Knowledge and Attitudes of Lebanese Mothers towards Dietary Supplement Practices in Maternal, Infancy and Preschool Ages: Findings of a National Representative Cross-Sectional Study
Source: Foods. 2022 Sep 27;11(19):3005. doi: 10.3390/foods11193005 (PMC9561973; doi:10.3390/foods11193005)
Supplement: Supplementary file 1 [file foods-11-03005-s001.zip › foods-1860376-supplementary.pdf]

Table S1. Mothers' knowledge, attitudes, and practices related to oral dietary supplementation.

Statement	Mothers (N=511)
Knowledge	n (%)
HPs can cause adverse effects when consumed with drugs	
No	382 (74.7)
Yes	129 (25.3)
HPs are safe because they are from natural sources (N=509 mothers)	
No	163 (32.0)
Yes	346 (68.0)
The effectiveness of DSs should be based on clinical trials	
No	242 (47.4)
Yes	269 (52.6)
DSs available at pharmacies are pretested for their safety (n=511 mothers)	
No	341 (66.7)
Yes	170 (33.3)
**Attitudes**	**n (%)**
DSs are necessary to maintain good health (n=502 mothers)	
Agree	311 (61.9)
Disagree	191 (38.1)
DSs help fight infections by strengthening immunity (n=504 mothers)	
Agree	295 (58.5)
Disagree	209 (41.5)
Food nutrients are sufficient to meet dietary requirements with no need for DSs (n=509 mothers)	
Agree	219 (43.0)
Disagree	290 (57.0)
DSs' consumption could cause health adverse effects (n=505 mothers)	
Agree	128 (25.4)
Disagree	377 (74.6)
DSs may mitigate the negative effects associated with smoking, alcohol consumption, and being sedentary (n=510 mothers)	
Agree	67 (13.2)
Disagree	443 (86.8)
**Practices**	**n (%)**
Use of DSs	
Yes	242 (47.4)
No	269 (52.6)
Type of supplement (n=242; supplement users)	
Vitamin D	163 (82)
Iron	162 (67)
Calcium	148 (61)
Multivitamin	136 (56)
Magnesium	114 (47)

Vitamin C	99 (41)
Vitamin B12	75 (31)
Zinc	73 (30)
Vitamin B6 (pyridoxine)	73 (30)
Folic acid	70 (29)
Vitamin E	46 (19)
Vitamin B1 (thiamin)	46 (19)
Vitamin A	44 (18)
Vitamin B2 (riboflavin)	39 (16)
Iodine	39 (16)
Niacin	36 (15)
Phosphorus	36 (15)
Choline	31 (13)
Selenium	31 (13)
Looking over the DS's label before using it (n=242 mothers)	
Most of the time	126 (52.0)
Occasionally	56 (23.1)
Rarely	44 (18.1)
Never	16 (6.6)
DSs are prescribed by (n=242 mothers)	
Physician	204 (84.2)
Dietitian	11 (4.5)
Coach (trainer)	3 (1.2)
Friend	1 (0.4)
Own willing (self-decision)	23 (9.5)
DSs are purchased from (n=242 mothers)	
Pharmacy	232 (95.9)
Nutrition supplements store	7 (2.9)
Online purchase	3 (1.2)
Supplement's form (n=242 mothers)	
Pills	234 (96.6)
Drinks	8 (3.3)
Money spent per month on purchasing DSs (n=239 mothers)	
Less than 50,000 L.L.	64 (26.7)
50,000-100,000 L.L.	117 (48.9)
100,000-200,000 L.L.	45 (18.8)
More than 200,000 L.L.	13 (5.4)
Adverse effects experienced after DSs use (n=242)	
Tachycardia	4 (1.7)
Nausea, with or without vomiting	7 (2.9)
Stomach pain	6 (2.5)
Headache	4 (1.7)
Dizziness and confusion	1 (0.4)
Chronic constipation	7 (2.9)
Skin itching	2 (0.8)

Table S2. Practices related to oral dietary supplementation among under- 5 children in this study.

Practices	Overall (N=511)		Boys (n=230)		Girls (n= 281)		*p*-Value
	N	%	n	%	n	%	
Use of DSs							
Yes	329	64.4	157	68.3	172	61.2	0.01*
No	182	35.6	73	31.7	109	38.8	
Type of supplement (n=329; supplement users)							
Single vitamin/or mineral	153	46.6	78	49.6	75	43.8	0.34
Multi-vitamin-mineral	110	33.2	53	33.5	57	33	
Single vitamin/or mineral and multi-vitamin-mineral	66	20.2	27	16.9	39	23.2	
The DSs are prescribed for the child by (n=329; supplement users)							
Physician	315	95.7	150	95.7	165	95.3	0.58
Pharmacist	13	4.0	7	4.3	6	3.6	
Mothers' own willing	1	0.3	0	0	1	0.8	
Reasons that led mothers to supplement their children's diet (n=329; supplement users)							
Keep the child healthy	197	59.8	84	53.5	113	66.1	0.17
Food nutrients are not enough to meet child's dietary needs	100	30.5	57	36.3	43	25.1	0.04*
The child has a health problem that necessitates DSs use	32	9.7	16	10.2	15	8.8	0.80

*significant at p-value <0.05 for χ 2 test.
